# Baicalein mitigates epithelial barrier impairment and microbiota dysbiosis in allergic asthmatic mice via the gut‑lung axis

**DOI:** 10.1186/s13020-026-01427-8

**Published:** 2026-05-21

**Authors:** Yao Lu, Xinqian Rong, Lini Wei, Jingwen Yang, Kaiqi Zhang, Yong Tan, Ning Zhao, Xiaojuan He, Cheng Lu, Li Li

**Affiliations:** 1 Institute of Basic Research in Clinical Medicine, China Academy of Chinese Medical Science, Beijing, 100010 People’s Republic of China; 2 Maoming Hospital, Guangzhou University of Chinese Medicine, Maoming, Guangdong 525000 People’s Republic of China; 3 Infectious & Respiratory Department, Ningbo Traditional Chinese Medicine Hospital Affiliated to Zhejiang Chinese Medical University, Ningbo, Zhejiang, 315012 People’s Republic of China

**Keywords:** Baicalein, Allergic asthma, *Akkermansia muciniphila*, Propionic acid, Epithelial injury

## Abstract

**Background:**

Allergic asthma (AA) may result in repeated episodes of chest constriction and coughing. In its most serious manifestations, it can cause death by asphyxiation. Currently, no efficacious therapeutic interventions exist to avert or counteract these serious outcomes. Baicalein (BAI) is a core quality marker of the traditional Chinese medicine *Scutellaria baicalensis*, but the mechanism of its oral action remains unclear.

**Objective:**

Assess the therapeutic efficacy of BAI in AA mice models and investigate its mechanism of action.

**Study design and methods:**

Evaluate the efficacy of BAI on ovalbumin-induced AA mice. To assess alterations in the pulmonary and gut microbial communities, 16S rRNA sequencing was employed. The integrity and restoration of the lung and intestinal epithelial lining were evaluated via immunohistochemistry. Furthermore, gas chromatography–mass spectrometry quantified fecal levels of short-chain fatty acids (SCFAs) in AA mice, and flow cytometry was used to analyze the content of ILC2 cells in colon tissue. Finally, the role of beneficial bacteria and their metabolites in inhibiting AA was further confirmed through fecal microbiota transplantation (FMT).

**Results:**

Oral BAI effectively alleviated AA-related lung epithelial damage and microbiota dysbiosis, while elevating the production of the tight junction proteins. Moreover, BAI mitigated colonic epithelial damage, inhibited ILC2s activation in the colon, enriched the abundance of gut probiotics capable of producing SCFAs, especially *Akkermansia muciniphila (A. muciniphila)*, and increased the content of SCFAs such as propionic acid in feces. The FMT experiment conducted after gavage with broad-spectrum antibiotics confirmed that BAI mediated reversal of microbial dysbiosis plays a key role in the treatment of AA, significantly increasing the expression of GPR41 mRNA in colon tissue and inhibiting the activation of ILC2s.

**Conclusion:**

The potential prebiotic BAI mitigates AA via targeting *A. muciniphila* and its metabolites, which consequently inhibits epithelial damage and type 2 immune activation.

**Graphical Abstract:**

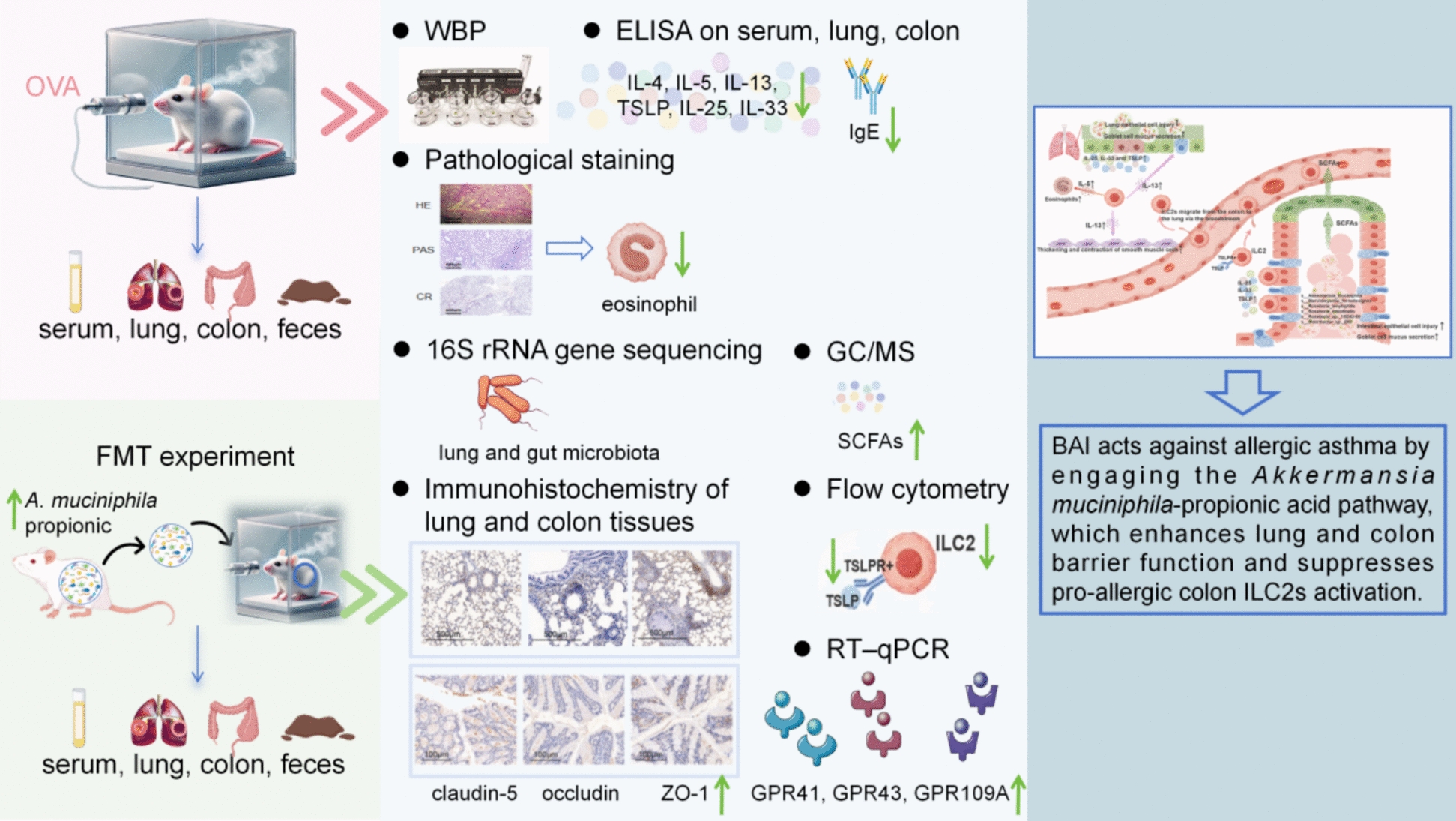

**Supplementary Information:**

The online version contains supplementary material available at 10.1186/s13020-026-01427-8.

## Introduction

Allergic asthma (AA), the most common phenotype of chronic inflammatory asthma, is defined by a type 2 immune response, clinically characterized by airway hyperresponsiveness (AHR), reversible airflow obstruction, excessive mucus secretion, inflammatory cytokine production, and eosinophil infiltration [1, 2]. Asthmatic patients exhibit damaged airway epithelial barriers, reduced tight junction (TJ) proteins, increased cellular permeability, and airway epithelial cells secreting large amounts of alarmins, including thymic stromal lymphopoietin (TSLP), interleukin-25 (IL-25), and interleukin-33 (IL-33) [3]. Consequently, type 2 innate lymphoid cells (ILC2s) are swiftly activated and produce a large amount of type 2 cytokines, collectively promoting inflammation in the airways [4]. Globally, the prevalence of asthma continues to increase, imposing a heavy health burden and economic pressure on society [5]. Current asthma treatment strategies primarily aim to reduce airway inflammation and promote bronchodilation, and first-line medications include corticosteroids and leukotriene receptor antagonists, among others [6]. However, some patients exhibit treatment resistance or face a significant risk of side effects [7]. Therefore, exploring novel, safe, and effective alternative or adjuvant therapeutic strategies with distinct mechanisms has become important.

The intestine can remotely regulate lung immune responses by regulating the microbiota and its metabolite homeostasis, mucosal barrier function, and local immunity [8]. Clinical studies have been proved that asthma patients are more likely to experience gastrointestinal discomfort [9] and that early development of the gut immune system can influence asthma onset [10]. Moreover, asthma development may be driven by the migration of gut immune cells (e.g., ILC2s) [11–15] and the production of inflammatory factors [16], which are often accompanied by microbial homeostasis imbalance [16, 17], gut epithelial barrier damage [18] and intestinal immune dysregulation [19]. Thus, intestinal barrier dysfunction, disruption of the gut microbial community, and the consequent chronic, low-level systemic inflammation are viewed as principal elements that initiate or worsen immune disorders like AA. Fecal microbiota transplantation (FMT) is one of the most commonly used methods for studying the causal relationship between gut microbiota and animal model diseases [20]. FMT shows that the transplanted microbiota can affect disease status and alleviate various diseases including acute respiratory distress syndrome [21], chronic obstructive pulmonary disease [22], inflammatory bowel disease [23] and asthma [24], etc. Nonetheless, research examining the gut-lung microbial axis and immune balance in individuals with AA remains limited. As a result, targeting the gastrointestinal tract to regulate respiratory immune responses has arisen as a novel and encouraging treatment strategy.

Contemporary research indicates that numerous constituents within conventional Chinese medicinal practices may reduce manifestations of respiratory illnesses through the regulation of intestinal microbiota balance and immunological activity [25]. Short-chain fatty acids (SCFAs) are mainly produced by the fermentation of dietary food by intestinal bacteria. As binding molecules for G protein-coupled receptors (GPCRs), they not only affect the host's intestinal microenvironment, but can also be absorbed into the bloodstream, systematically affecting the immune status of the body, thereby reducing lung tissue damage and regulating type 2 immune response [26–28]. Research has shown that the abundance of beneficial bacteria producing SCFAs in the intestines of asthma patients decreases, and the concentration of SCFAs in feces and blood also decreases, leading to damage to the epithelial cell barrier in the lungs and intestines and activation of multiple pro-inflammatory pathways [27, 29]. Therefore, metabolic disorders, gut microbiota dysbiosis with intestinal barrier damage, systemic chronic inflammation, and pulmonary inflammation with airway hyperresponsiveness interweave into a self reinforcing cycle, synergistically amplifying the progression of AA. Derived from the roots of *Scutellaria baicalensis*, baicalein (BAI) is a principal flavonoid with documented anti-inflammatory, anti-allergic, and immune-regulating characteristics, leading to its extensive historical use [30]. BAI has been demonstrated to have efficacy on asthma [31–35] and treat respiratory diseases by regulating the intestinal microenvironment [36]. This compound can supply fuel to particular bacterial populations residing in the gut, producing SCFAs molecules [36], and can also improve colitis by inhibiting intestinal immune-inflammatory responses, reducing colonic permeability, and restoring tight junctions [32]. This study aimed to evaluate the pivotal role of BAI in alleviating airway and intestinal epithelial injury in AA mice by maintaining intestinal microbial homeostasis and regulating metabolites. Oral intervention with BAI was performed on OVA-induced AA mouse models. 16S rRNA sequencing was used to analyze structural changes in the lung and gut microbiota; immunohistochemistry was adopted to assess the integrity and repair of the epithelial barrier; targeted metabolomics was applied to detect the levels of SCFAs; and flow cytometry was utilized to determine the activation status of ILC2s. Finally, FMT was conducted to verify the causal relationship between BAI-mediated beneficial gut bacteria and their metabolites in inhibiting epithelial injury associated with allergic asthma.

## Materials and methods

### Chemicals and reagents

The reagents applied in the present experiments included ovalbumin (OVA; A5503, Sigma, USA), aluminium hydroxide adjuvant (77161, Thermo, USA), baicalein obtained from Aladdin (B107324, Shanghai, China), acetyl-β-methylcholine chloride (A2251, Sigma, USA), and montelukast (T1677, TaoShu, Shanghai, China). Enzyme-linked immunosorbent assay kits specific for IL-4 (MU30385), IL-5 (MU30011), IL-13 (MU30012), TSLP (MU30422), IL-25 (MU30777), and IL-33 (MU30762) were supplied by Beyinlai (Wuhan, China). Primary antibodies targeting claudin-5 (PAB57427, Beyinlai, Wuhan, China), occludin (27260-1-AP, Proteintech, Wuhan, China), and ZO-1 (GB15195, Servier, Wuhan, China) were used for subsequent analyses.

### Establishing OVA-induced AA mice and medical treatment

6–8-week-old female SPF BALB/c mice were purchased from Beijing Weitonglihua Experimental Animal Technology Co., Ltd.. Standard rodent diet and water were available without restriction. The handling of subjects and all methodological protocols followed the ARRIVE guidelines and received approval from the Animal Ethics Committee of the China Academy of Chinese Medical Sciences (No.: IBTCMCACMS21-2308-03).

**Fig. 1:**
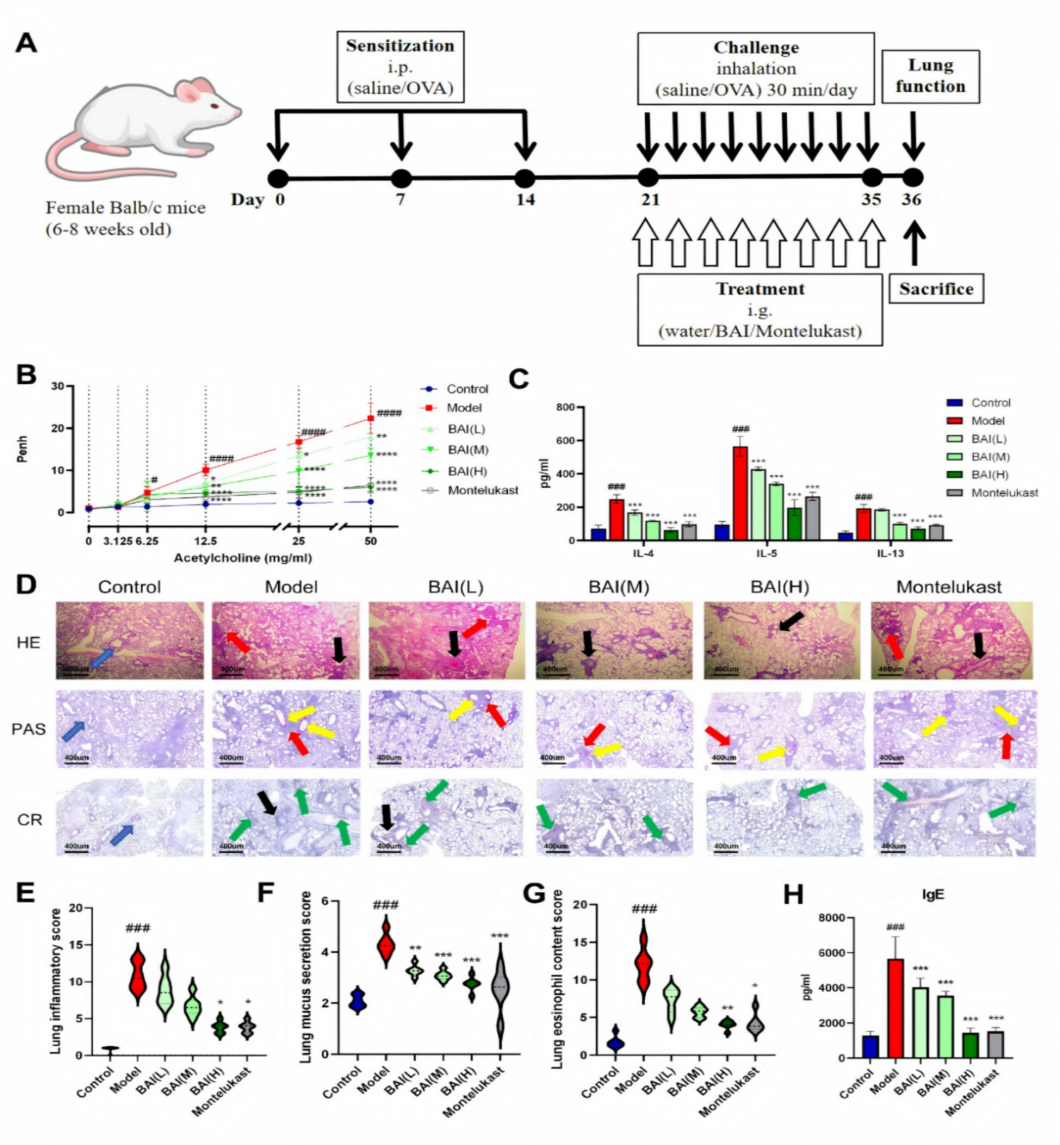
BAI alleviates the pulmonary inflammatory response and AHR in AA mice. **A** Experimental protocol and drug therapy for OVA induced AA mouse model. **B** Penh values (n = 4). **C** Levels of inflammatory factors in lung tissue (n = 6). **D**–**G** Typical lung tissue staining image. Scale bar: 400 µm; original magnification: 100×. The blue arrow represents the normal structure of lung tissue. The red arrows indicate the presence of a large amount of inflammatory cell infiltration in the bronchial and alveolar cavities. The black arrows indicate significant thickening of the smooth muscles in the trachea and bronchi. The yellow arrow indicates an increase in mucus secretion. The green arrow indicates extensive infiltration of eosinophils. **H** Serum IgE levels (n = 6). Data are means ± SDs. #*P* < 0.05, ##*P* < 0.01, ###*P* < 0.001, ####*P* < 0.0001 versus the control group; *P < 0.05, **P < 0.01, ***P < 0.001 versus the model group

To induce an asthma model, rodents received intraperitoneal sensitization using 0.2 ml of a saline solution comprising 20 µg of OVA combined with 2 mg of AL(OH)_3_, administered on days 0, 7, and 14. Subsequently, from Day 21 to Day 35, the animals were exposed to physiological saline containing 1% OVA for 40 min per day via nebulization. During the challenge period, mice in the treatment groups received BAI at doses of 10, 20, and 40 mg/kg, or 10 mg/kg of montelukast by oral gavage, once per day. Montelukast, a clinically established leukotriene receptor antagonist widely prescribed for asthma management [37]. Other groups of mice received the same volume of sterile physiological saline solution (Fig. 1A).

### Faecal microbiota transplantation

FMT was conducted following standardized methods [20]. Faecal samples from the control, model, and BAI(H) groups were collected daily, pooleds [21]. Homogenize and centrifuge 100 mg of fecal sample in 1 ml of PBS containing 20% glycerol (2000 g, 1 min) to remove particulate matter [22]. To reduce commensal bacterial populations, the recipient rodents received an oral mixture of wide-spectrum antimicrobial agents, consisting of vancomycin (100 mg·kg^−1^), neomycin sulfate (200 mg·kg^−1^), metronidazole (200 mg·kg^−1^), and ampicillin (200 mg·kg^−1^) [38]. Absolute qPCR confirms complete depletion of endogenous gut microbiome in recipient mice. During Day 21 to Day 35, the recipient mice in each group were gavaged with 200 µl of the prepared faecal microbiota suspension every other day [39] (Fig. 5A).

### Histopathological analysis

Specimens of right lung and distal colon were fixed in paraformaldehyde, processed through embedding, sectioning, and staining [40]. Subsequently, all tissue slices were imaged with a slide scanner (Leica, Aperio CS2, USA). Following channel separation of the digital images, regions of interest were delineated through threshold-based segmentation. The findings from the optical density assessment are presented as the proportion of the stained region (threshold area) relative to the entire analyzed field.

### Whole-body plethysmography (WBP) to assess airway reactivity

WBP positioned the mice, and AHR was reflected by the enhanced pause (Penh) value. Baseline values for each group were adjusted using PBS, and the baseline recording lasted approximately 2 min. The mice were then challenged by nebulization with acetyl-β-methylcholine chloride (Mch) at concentrations of 0–50.00 mg·mL^−1^. Each nebulization lasted 30 s, after which the Penh value was recorded for 3 min. The average Penh value over the 3-min period was calculated. A higher Penh value indicates greater airway resistance and more severe airway narrowing.

### ELISA

Wash the lung and colon tissue samples with PBS (0.01 M, pH 7.4) to remove residual blood or surface impurities. Then weigh, record, and cut the tissues into small pieces. Add pre-cooled PBS and protease inhibitors to the tissue at a specified ratio, and homogenize the mixture. Centrifuge the homogenate at 4 °C and 10,000 × g for 5–10 min, and collect the supernatant. Following the instructions of the ELISA kit, measure the levels of IL‑4, IL‑5, IL‑13, IL‑25, IL‑33, and TSLP in the lung tissue; IL‑25, IL‑33, and TSLP in the colon tissue; and IgE levels in the serum.

### Immunohistochemical analysis

Treat paraffin sections with xylene, and then rehydrate with a series of ethanol solutions. Following a 15-min heat-induced antigen retrieval in citrate buffer at 95 °C, a peroxidase blocking agent was used for 30 min. A subsequent PBS wash preceded a 30-min incubation with normal serum to block nonspecific binding. Next, the tissue slices were exposed for one hour at 37 °C to primary antibodies targeting claudin-5, ZO-1, and occludin. The dilution ratios are 1:800, 1:1200, and 1:200, respectively. Add HRP labeled goat anti rabbit IgG diluted at a ratio of 1:200 and incubate for 30 min. To assess the level of staining, three arbitrary visual areas from each sample were chosen for analysis of relative signal intensity.

### Flow cytometry

Detection of ILC2s (CD45⁺Lineage⁻CD127⁺CD90.2⁺GATA3⁺) and TSLPR content in colon tissue. Total cell counts were obtained using a hemacytometer. Add 100 μL of stimulant (including RPMI-1640 complete culture medium and Cell Activation Cocktail) to each cell sample. Add 100 μL of surface antibody mixture (containing the live or dead dye Zombie Aqua and surface marker antibodies such as CD45, TSLPR, Lineage, CD90.2, CD127) for surface staining. Add fixative, lysis buffer, and intracellular antibody mixture (containing GATA3 antibody) in sequence. Before boarding, monochromatic compensation microspheres were used for fluorescence compensation adjustment, followed by detection using a flow cytometer (CytoFlex S).

### RT‒qPCR analysis

RNA isolation was carried out on colonic samples with TRIzol™. Convert the isolated RNA into cDNA, designed for qPCR with genomic DNA elimination (TransGen, Beijing, China). Subsequent qPCR assays were carried out with PerfectStart® Green qPCR SuperMix to measure the expression of GPR41, GPR43, and GPR109A. GAPDH functioned as the internal control gene for normalization (Table 1). Sangon Biotech (Shanghai, China) provided all oligonucleotide primers. The LightCycler® 480 Software was utilized to run 50 amplification and quantification cycles. Target gene expression, relative to GAPDH, was determined via the 2⁻ΔΔCT calculation.

**Table 1:** Primer sequences used for quantitative polymerase chain reaction

Gene	Forward Primer sequence (5′−3′)	Reverse Primer sequence (5′−3′)
GPR41	GCCACACTGCTCATCTTCTTCG	CGCCAGGACGGACTCTCAC
GPR43	CGGGTTCGCCAGCCTCAG	CAGCAGCAACAGGAGCAAGTC
GPR109A	CCTGACTGTCCACCTCCTCTATAC	ATCGTGCCACCTGAAGTT GTAAC
GAPDH	TGGTGAAGCAGGCATCTGAG	TGCTGTTGAAGTCGCAGGAG

### Absolute qPCR analysis

Genomic DNA was isolated from the fecal specimens collected from every cohort, with its quality and completeness subsequently evaluated. Use purified DNA as a template for quantitative PCR targeting the V4 hypervariable segment within the bacterial gene. Amplification of the 289 bp V4 region was performed with the universal primer pair V4F_517_17 and V4R_805_19 [41]. A NanoDrop 2000 spectrophotometer (Nucliber) was employed to determine plasmid concentration, after which the plasmid copy number was derived from its molecular weight. To estimate the quantity of bacteria present in individual samples, the plasmids were diluted 500-fold and then serially diluted tenfold with ddH₂O to create an 8-point standard curve template from n10^1^⁰ to n10^3^ copies/µl. A calibration curve was constructed from reference materials containing predetermined copy numbers. The initial concentration of unknown specimens was then determined based on their Ct values using this curve, which enabled the absolute measurement of mRNA or DNA molecule quantities within the analyzed samples.

### Microbiome analysis

Genomic DNA was isolated from all mouse lung and fecal samples for subsequent characterization of microbial communities. This purified DNA served as the template to target and amplify the hypervariable segments from V1 to V9 of the 16S ribosomal DNA for complete-length sequencing, employing the broad-range primers 27F and 1492R, after which sequencing libraries were prepared. Amplicon sequence variants (ASVs) were then clustered on the basis of 100% similarity using the effective data. Representative sequences of ASVs were annotated for species using the Greengenes database (version 13.5). Following the ASV clustering outcomes, microbial diversity was assessed with QIIME 2. On the basis of the species annotation results, species composition information at various taxonomic levels was obtained. High-throughput sequencing was completed by Shanghai Majorbio Biopharm Technology Co., Ltd.

### Determination of Faecal SCFAs

Samples were homogenized in a mixture of phosphoric acid, internal standard, and ether and centrifuged at 12,000 rpm for 10 min. Following filtration using a porous membrane, the resulting supernatant was collected for subsequent analysis. An aliquot of 1 µL was introduced into the GC–MS system. Quantification of multiple short-chain fatty acid levels was carried out via monitoring of selected ion and made a determination of by the approach of internal standard.

### Statistical analysis

Data are presented as the mean ± standard deviations (SDs) and were analyzed using GraphPad Prism 9 (GraphPad Software, CA, USA). For comparisons among multiple groups, statistical analysis was conducted using either one-way or two-way ANOVA. The P value equal to or below 0.05 served as the threshold for establishing statistical significance.

## Results

### BAI attenuates AHR and inflammatory responses in a murine model of AA

BAI alleviated AHR in allergic asthma mice. The response to Mch serves as a key method for quantifying the AHR that commonly results from allergic airway inflammation. Therefore, on Day 36, mice were exposed to escalating concentrations of Mch (0–50 mg/mL). The Penh response to Mch was significantly elevated in AA mice compared to controls (*P* < 0.0001) (Fig. 1B), consistent with successful AHR induction via OVA sensitization and challenge. Treatment with 40 mg/kg BAI significantly reduced AHR (*P* < 0.0001).

BAI reduced lung inflammation and pathological status in mice with allergic asthma. The lung index, serum IgE, and levels of related inflammatory factors (IL-4, IL-5, and IL-13) in the model group were significantly increased (*P* < 0.001), while the BAI(H) group showed a significant decrease (*P* < 0.001) (Figs. 1C, H, S1). The model group showed significant pathological changes, including abnormal alveolar structure, thickening of alveolar walls, extensive eosinophils infiltration, increased mucus secretion, and increased bronchial smooth muscle thickness. BAI treatment significantly reduced this infiltration (Fig. 1D–G).

### BAI modulates the lung microbiota and alleviates lung epithelial injury in AA mice

**Fig. 2:**
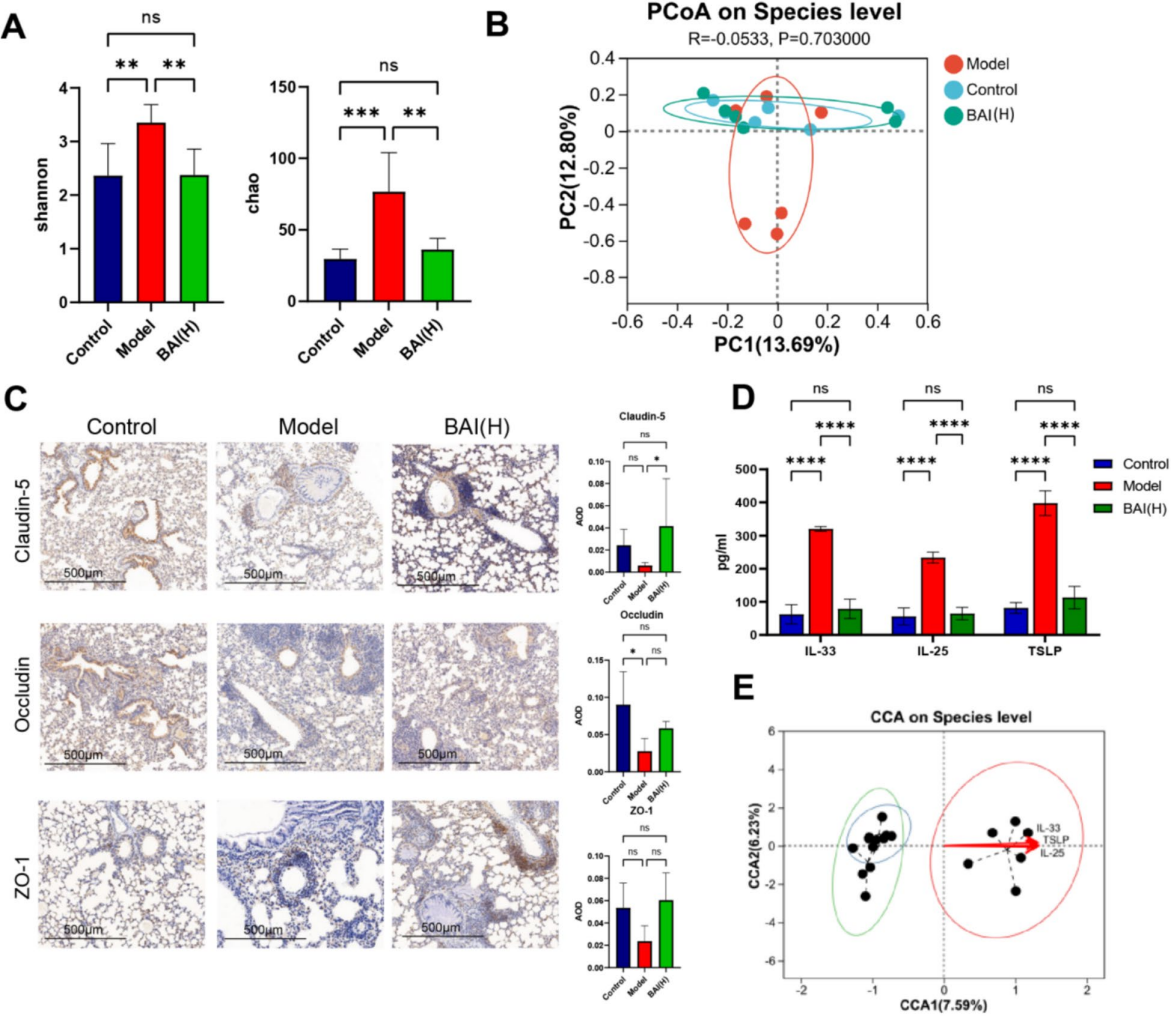
BAI restores lung microbiota and reduces pulmonary epithelial injury. **A** α-Diversity analysis (n = 6). **B** PCoA (n = 6). **C** Representative immunohistochemical staining images showing TJ proteins expression (n = 4). Scale bar: 500 µm; original magnification: 100×. AOD quantitative analysis of relative expression of TJ proteins. **D** Levels of alarmins in lung tissue (n = 6). **E** RDA/CCA of alarmins and bacterial species. Data are means ± SDs. *P < 0.05, **P < 0.01, ***P < 0.001, ****P < 0.0001

Maintaining lung microbial homeostasis, preserving the lung epithelial barrier, and reducing inflammatory factor secretion from lung epithelial cells are key to protecting the lung epithelium. The α-diversity (Shannon and Chao indices) of the lung tissue microbiota significantly increased in the model group (*P* < 0.01, *P* < 0.001). In contrast, BAI treatment significantly reduced these indices (*P* < 0.01) to levels comparable to the control. Furthermore, β-diversity was assessed via PCoA based on the abund_jaccard distance. Distinct clustering patterns were observed, with the control and BAI(H) groups clustering more closely together than the model group did (Fig. 2 A, B). Similarly, the heatmap showed that compared with the model group, the relative abundance of *g_Alistipes* and other bacteria in lung tissue was decreased after BAI treatment (Fig. S2). BAI treatment significantly caused an upward trend or significant increase in the expression of TJ proteins in lung tissue (*P* < 0.05) (Fig. 2C) and reduced lung tissue levels of alarmins (*P* < 0.0001) (Fig. 2D). And the molecular docking fraction range between BAI and alarm protein is − 6.4 to − 7.8 kcal/mol, with strong binding strength and the ability to form stable spatial structures (Fig. S3). Moreover, the RDA/CCA results revealed a positive correlation between the model group and the expression of alarmins (Fig. 2E).

### BAI increases the abundance of *A. muciniphila* and the propionic acid content in faecal samples

**Fig. 3:**
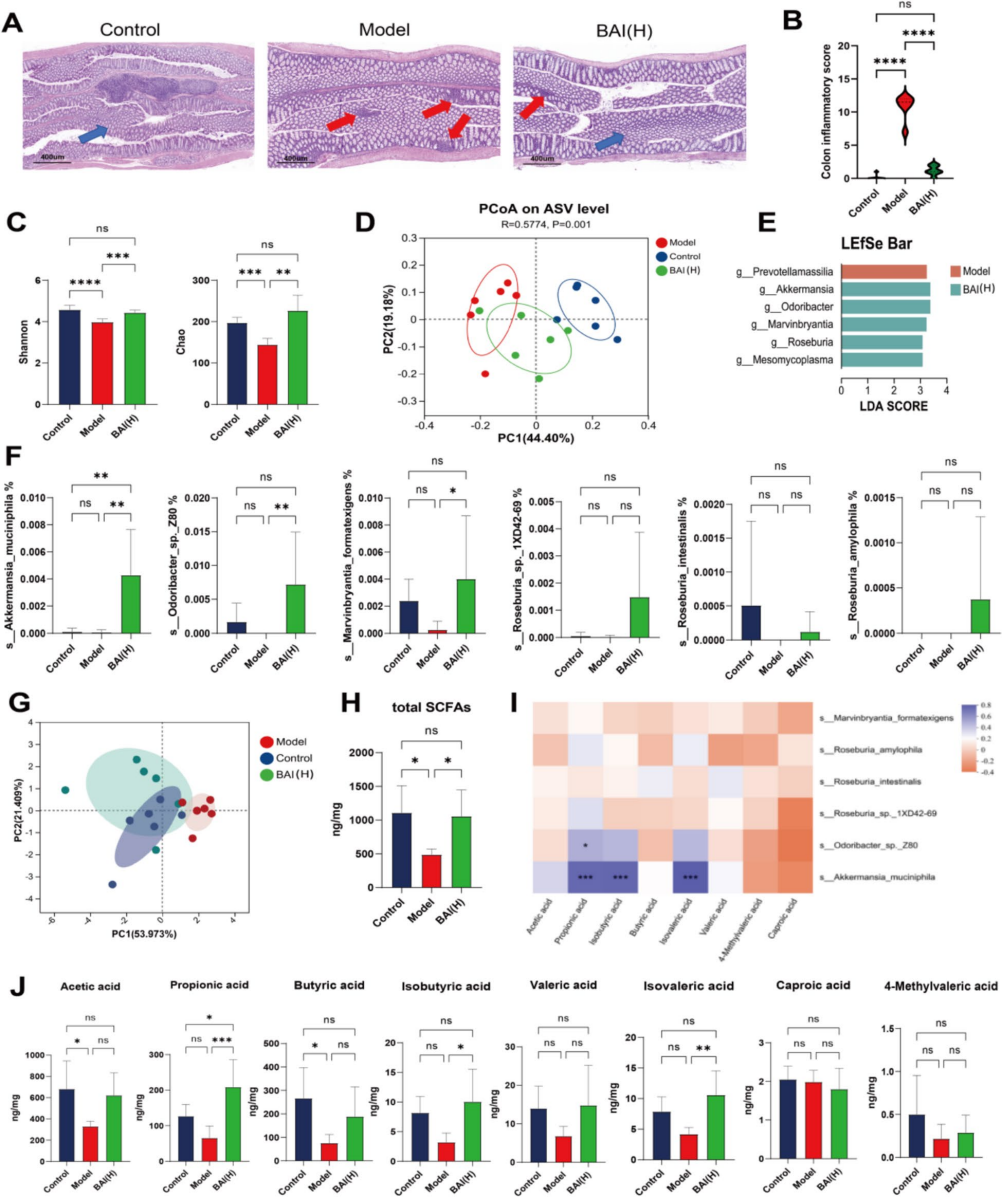
BAI alleviates the colonic inflammatory response and microbial dysbiosis in AA mice. **A**, **B** Representative staining images of colon tissue. Scale bar: 400 µm; original magnification: 100×. The blue arrow represents the normal structure of colon tissue. The red arrow indicates inflammatory cell infiltration. **C** α-Diversity analysis (n = 6). **D** PCoA (n = 6). **E** Taxonomic abundance generated by LEfSe analysis (n = 6). **F** Relative abundance of dominant bacterial species after BAI treatment (n = 6). **G**, **H**, **J** Contents of SCFAs in the colon (n = 6). **I** Pearson correlation analysis between the expression of dominant bacterial species and SCFA levels after BAI treatment (n = 6). Data are means ± SDs. *P < 0.05, **P < 0.01, ***P < 0.001, ****P < 0.0001

In model group, partial inflammatory infiltration in the intestine, abnormal crypt structure in some areas, visible abscesses in some areas, disordered gland arrangement, and damage to individual glands were observed. In BAI(H) group, the degree of inflammatory infiltration in the intestinal tissue submucosa decreased, the crypt structure recovered, abscess severity decreased, and gland arrangement was restored (Fig. 3A, B). Results of α-Diversity analysis revealed that the Shannon and Chao indices of the faecal microbiota were markedly reduced in the model group (*P* < 0.0001, *P* < 0.001), while these indices were notably elevated following BAI treatment (*P* < 0.001, *P* < 0.01) (Fig. 3C). PCoA analysis observed that the clustering of the control group and BAI(H) group was tighter (Fig. 3D). Using LEfSe analysis, we pinpointed the dominant bacterial genera in the BAI(H) group relative to those in the model group, including *g_Akkermansia*, *g_Odoribacter*, *g_Marvinbryantia*, *g_Roseburia*, and *g_Mesomycoplasma* (Fig. 3E). Notably, the top 4 genera all have the ability to produce SCFAs, including *s_Marvinbryantia_formatexigens*, *s_Roseburia_amylophila*, *s_Roseburia_intestinalis*, *s_Roseburia_sp._1XD42-69*, *s_Odoribacter_sp._Z80*, and *s_Akkermansia_muciniphila*. The abundance of *s_A. muciniphila* was was notably greater in the BAI(H) group relative to both the control and model groups (*P* < 0.01, *P* < 0.01) (Fig. 3F), and this is consistent with the enrichment level of *A. muciniphila* in the fecal specimens of normal individuals and asthma patients shown in the GMrepo database (Fig. S4). Given that the dominant bacteria in the BAI(H) group generally have SCFA-producing capabilities, targeted metabolomics for SCFAs was performed on the faecal samples. Combined PCA and total SCFA content analysis showed significantly reduced SCFAs in the model group, which were markedly increased after BAI treatment, demonstrating its restorative effect (*P* < 0.05) (Fig. 3G, H). Interrelation analysis between SCFAs and 6 bacterial strains revealed strong positive correlations between *s_A. muciniphila* and propionic acid, isobutyric acid, and isovaleric acid (*P* < 0.001). Specifically, a marked increase in propionic acid content was observed in the BAI(H) group relative to the remaining groups (Fig. 3I, J).

### BAI alleviates colonic epithelial injury and inflammation in AA mice

**Fig. 4:**
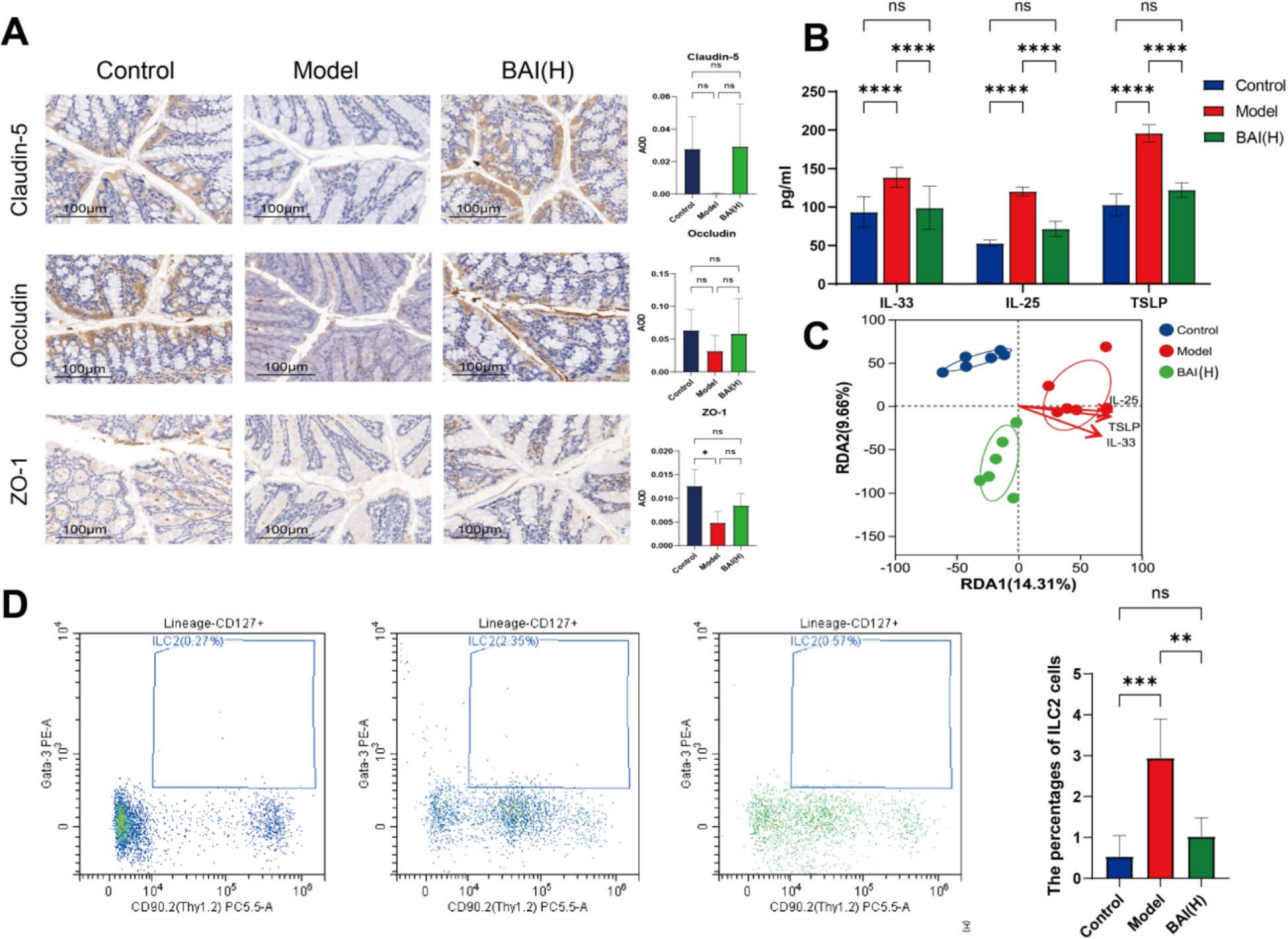
BAI reduces colonic epithelial injury and suppresses ILC2 activation. **A** Representative immunohistochemical staining images showing TJ proteins expression (n = 4). Scale bar: 100 µm; original magnification: 200×. AOD quantitative analysis of relative expression of TJ proteins. **B** Levels of alarmins in colon tissue (n = 6). **C** RDA/CCA of alarmins and bacterial species. **D** Proportion of ILC2s in mouse colon tissue. Data are means ± SDs. *P < 0.05, **P < 0.01, ***P < 0.001, ****P < 0.0001

Compromise of the intestinal epithelial barrier plays a significant role in AA pathogenesis [42]. Immunohistochemistry revealed that TJ protein expression in the model group was diminished, but this effect was reversed by BAI treatment (Fig. 4A). Epithelial barrier damage is often accompanied by the secretion of inflammatory factors from epithelial cells [43].The levels of alarmins were significantly increased in AA mice (*P* < 0.0001), and after BAI treatment, all the indicators significantly decreased (*P* < 0.0001) (Fig. 4B). The RDA/CCA analysis results of gut microbiota are consistent with the lung results (Fig. 4C). The BAI(H) group significantly reduced the number of ILC2sand TSLPR in the colon tissue (*P* < 0.01) (Fig. S5). Therefore, BAI plays a mitigating role in colonic epithelial barrier damage and inflammation.

### Faecal microbiota from BAI-treated donor mice attenuates lung epithelial injury in AA mice

**Fig. 5:**
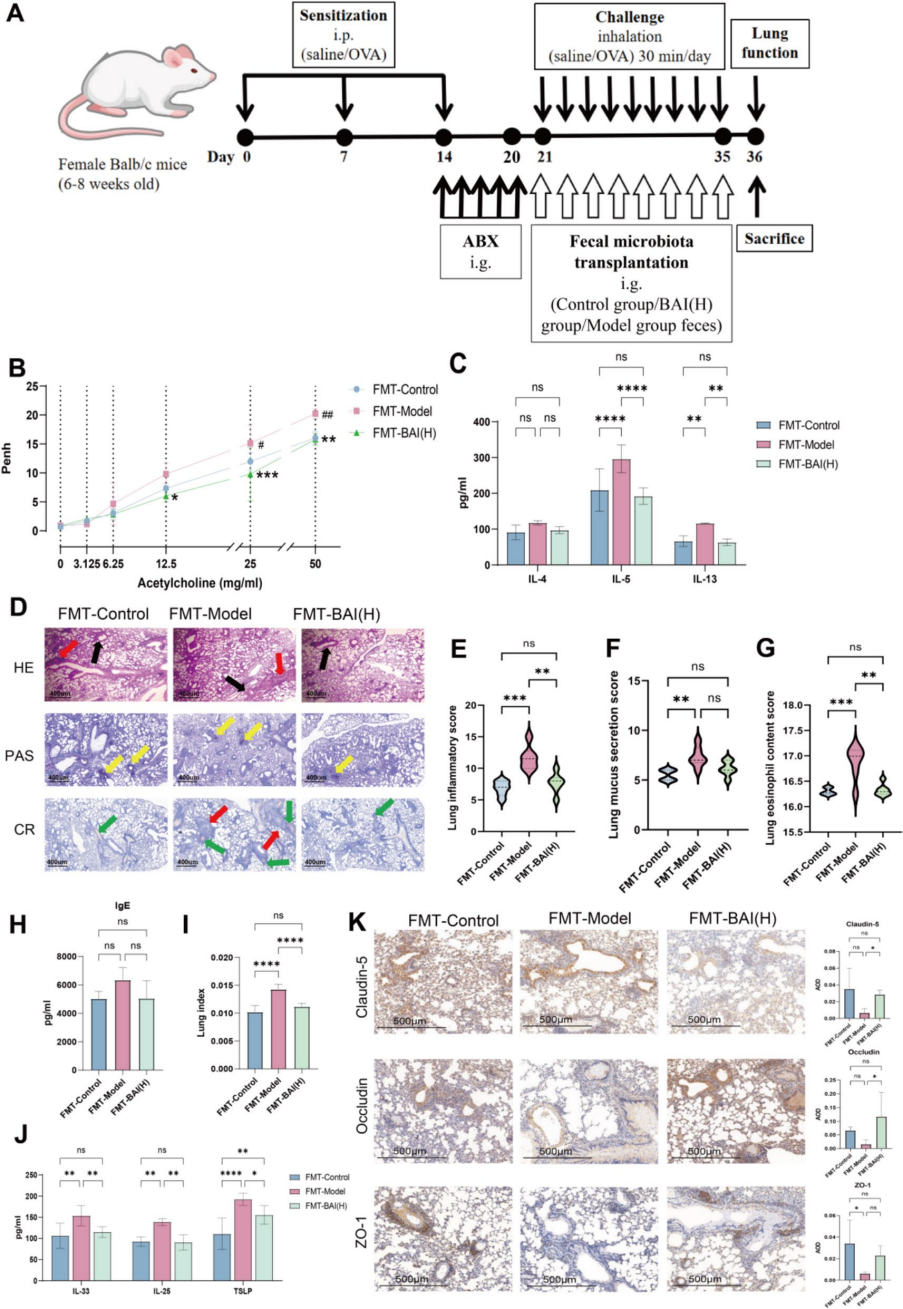
Faecal microbiota from BAI-treated donor mice alleviates pulmonary inflammation and epithelial damage in AA mice. **A** Experimental protocol for FMT. **B** Penh values (n = 4). **C** Levels of alarmins in lung tissue (n = 6). **D**–**G** Representative staining images of lung tissue. Scale bar: 400 µm; original magnification: 100×. The red arrows indicate the presence of a large amount of inflammatory cell infiltration in the bronchial and alveolar cavities. The black arrows indicate significant thickening of the smooth muscles in the trachea and bronchi. The yellow arrow indicates an increase in mucus secretion. The green arrow indicates extensive infiltration of eosinophils. **H** Serum IgE levels (n = 6). **I** Lung index. **J** Levels of alarmins in lung tissue (n = 6). **K** Representative immunohistochemical staining images showing TJ proteins expression (n = 4). Scale bar: 500 µm; original magnification: 200×. AOD quantitative analysis of relative expression of TJ proteins. Data are means ± SDs. *P < 0.05, **P < 0.01, ***P < 0.001, ****P < 0.0001

To further demonstrate that BAI affects allergic asthma through the gut microbiota, transfer the feces of the control group, model group, and BAI (H) group mice orally into the corresponding group (Fig. 5A). Transplantation of faecal material from the control and BAI (H) groups significantly alleviated AHR (*P* < 0.01) (Fig. 5B) and the relative levels of IL-5 and IL-13 in lung tissues (*P* < 0.0001, *P* < 0.01), but there was no significant difference in the relative level of IL-4 and serum IgE (Fig. 5C, H). Furthermore, lung histopathological examination revealed that the FMT-BAI(H) group had significantly reversed lesions, reduced inflammation severity and mucus secretion, and alleviated eosinophil infiltration (Fig. 5D–G). The lung index and alarmins was significantly lower in the FMT-Control and FMT-BAI(H) groups (*P* < 0.0001) (Fig. 5I–K), and enhanced TJ protein expression. In summary, BAI can maintain the disordered microbial environment in OVA-induced mouse lungs, protect the damaged lung epithelial barrier, and alleviate epithelial inflammatory injury.

### Faecal microbiota from BAI-treated donor mice attenuates intestinal inflammation and increases *A. muciniphila* abundance in AA mice

**Fig. 6:**
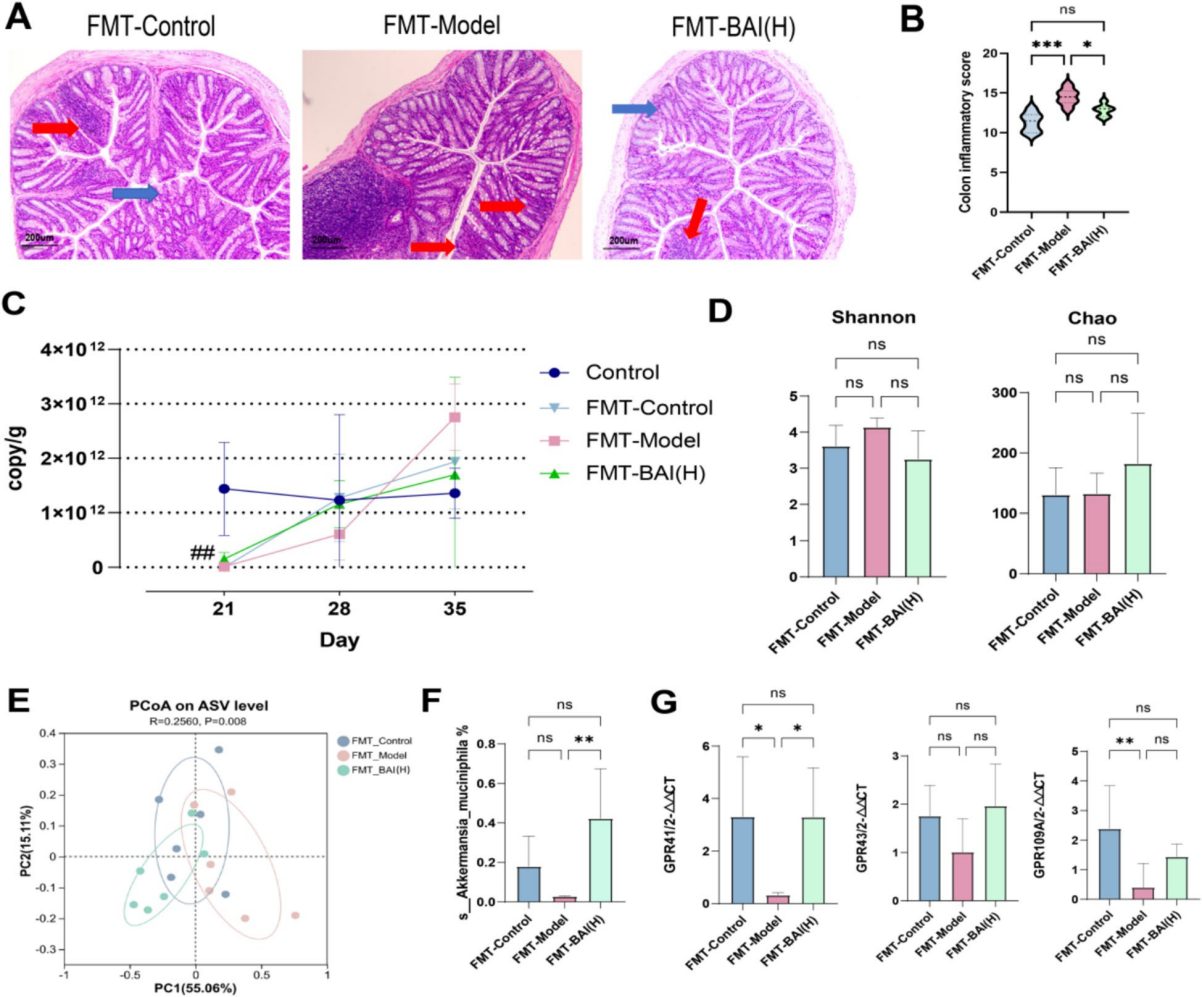
Faecal microbiota from BAI-treated donor mice alleviates the colonic inflammatory response and microbial dysbiosis in AA mice. **A**, **B** Representative images. Scale bar: 200 µm; original magnification: 100×. The blue arrow represents the normal structure of colon tissue. The red arrow indicates inflammatory cell infiltration. **C** Bacterial content in faeces. **D** α-Diversity analysis (n = 6). **E** PCoA (n = 6). **F** Relative abundance of A. muciniphila (n = 6). **G** Relative mRNA expression in the colon (n = 6). Data are means ± SDs. *P < 0.05, **P < 0.01, ***P < 0.001

Histopathological staining showed that the FMT-Control group and FMT-BAI(H) group had a reduced degree of submucosal inflammation infiltration in the intestinal tissue, restored crypt structure, reduced severity of abscess, and restored glandular arrangement (Fig. 6A, B). Day21 displayed the gut bacterial content in the faecal samples from the groups receiving broad-spectrum antibiotics decreased by 1000-fold (*P* < 0.01) and recovered to some extent after two weeks (Fig. 6C). The abundance of *s_A. muciniphila* in the FMT-BAI(H) group significantly increased (*P* < 0.01), suggesting that transplantation of the BAI donor faecal microbiota significantly enhanced the colonization of this bacterium in the recipient intestines (Fig. 6F). RT-qPCR measurement of GPR41, GPR43, and GPR109A mRNA in colon tissues showed that GPR41 expression was significantly elevated in the FMT-BAI(H) group (Fig. 6G).

### Faecal microbiota from BAI-treated donor mice protects the colonic epithelial barrier and alleviates inflammation in AA mice

**Fig. 7:**
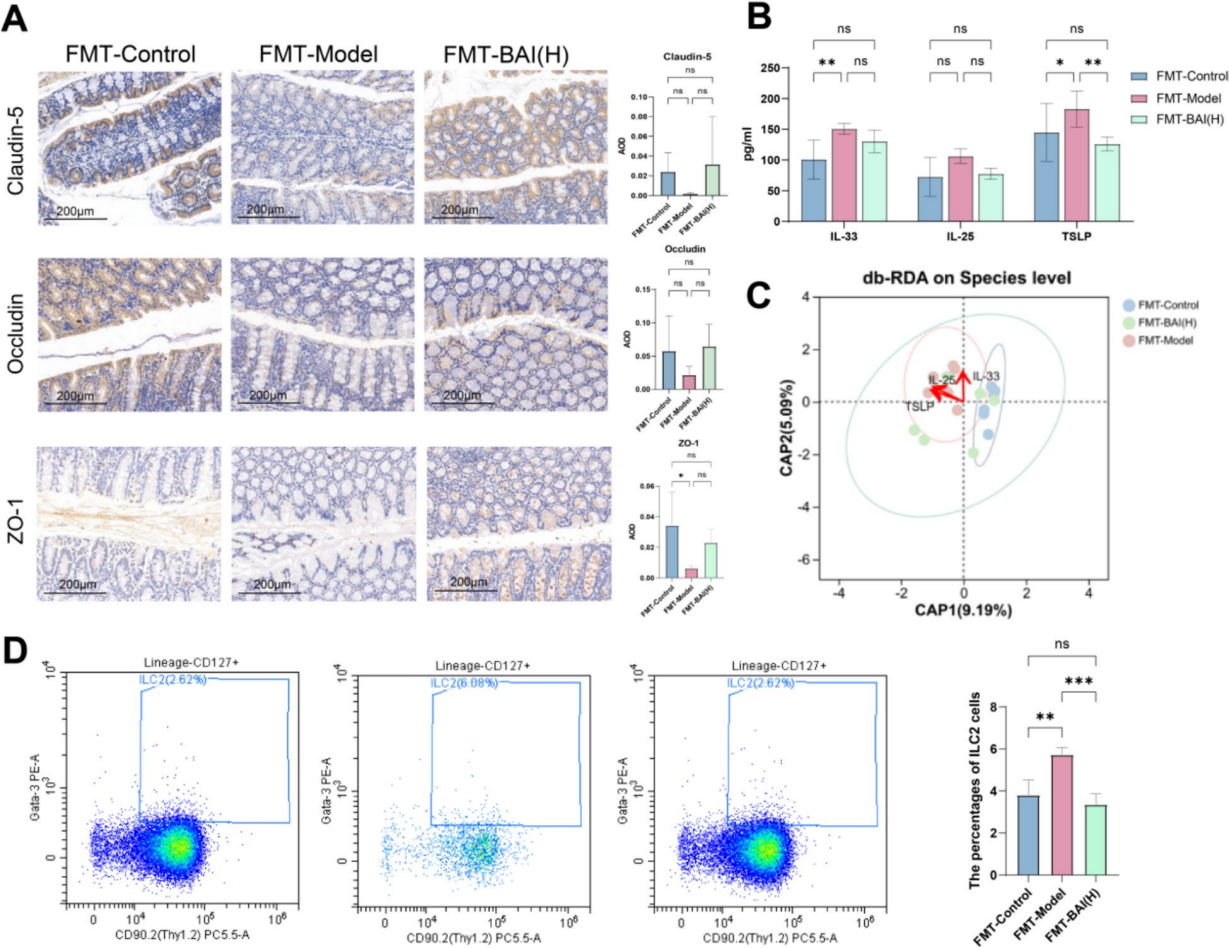
The fecal microbiota of BAI donors reduced colonic epithelial damage and inhibited ILC2 cell activation. **A** Representative immunohistochemical staining images showing TJ proteins expression (n = 4). Scale bar: 200 µm; original magnification: 200 × . AOD quantitative analysis of relative expression of TJ proteins. **B** Alarmins levels in colon tissue (n = 6). **C** RDA/CCA analysis of alarmins and bacterial strains. **D** Proportion of ILC2s in mouse colon tissue. Data are means ± SDs. *P < 0.05, **P < 0.01, ***P < 0.001。

The expression of TJ protein was upregulated and the levels of inflammatory factors were reduced in the FMT-Control group and FMT-BAI(H) group (Fig. 7A, B). The results of RDA/CCA for gut microbiota, the content of ILC2s and TSLPR in colon tissue are consistent with previous results (Figs. 7C–E, S6).

## Discussion

In AA, both the lung and intestine exhibit epithelial damage and disrupted microbial homeostasis, while gut microbiota metabolites and inflammatory cells can regulate the gut-lung interconnection [9, 13, 32, 36]. The TCM theory of “the lung and the large intestine are in harmony with each other” confirms the mutual communication and influence between organs in the human body. In this study, the pharmacological effects of BAI on AA mice were evaluated from the perspective of the “gut–lung axis”, and its mechanism of action concerning the protection against lung–gut epithelial damage and microbial homeostasis was explored.

A healthy epithelial barrier and microbial homeostasis play an important protective role in the human body, leading to epithelial cell damage and secretion of inflammatory factors under pathological conditions. The TJ proteins are core molecules that maintain epithelial barrier function [44]. Their disruption can lead to increased permeability [45]. Epithelial-secreted alarmins IL-25, IL-33, and TSLP, are brought to an upregulated state in innate immune responses to allergens in both the lung and the gut, and make an action upon innate and adaptive immune cells such as ILC2s and type 2 helper T cells [46–48]. Studies have shown that ILC2s can egress from the gut during intestinal infection, enter lymphatic vessels and blood, accumulate in the lungs as activated innate immune cells, and drive pulmonary inflammation, providing direct cellular immunological evidence for the modern scientific connotation of the “gut–lung axis” [12, 46, 47]. This study confirmed that BAI administration inhibits the activation of ILC2s in colonic tissues of AA mice. However, migration tracking experiments were not performed, and the direct correlation between ILC2s and AA has not been verified. Therefore, this mechanism remains speculative.

Pulmonary inflammation is reflected in the activation and secretion of inflammatory factors. IL-5 in lung tissue activates and recruits eosinophils, while IL-4 and IL-13 stimulate B cells to produce IgE, affecting airway epithelial cells and promoting AHR [49]. The study demonstrated that BAI not only lowered serum IgE and pulmonary inflammatory factors but also mitigated eosinophil infiltration and mucus hypersecretion, while concurrently enhancing tight junction protein expression. These findings suggest BAI may ostensibly take direct action on airway epithelial cells, whereupon it blocks the abnormal activation of type 2 immune responses. Additionally, BAI inhibited colonic epithelial damag and upregulated TJ protein expression.

Gut-lung microbiota dysbiosis, which directly impairs epithelial function, is a significant risk factor for AA [50]. BAI treatment exerted divergent effects on microbiota in AA mice, diminishing their abundance and diversity in the lungs but restoring them in the gut. This indicates that BAI may exert its therapeutic effects by restoring the balance of pulmonary and intestinal microbiota. Among the dominant bacterial genera in the faeces of BAI(H) group mice, *g_Akkermansia*, *g_Odoribacter*, *g_Marvinbryantia*, and *g_Roseburia* are capable of producing SCFAs [51–53]. *A. muciniphila* is believed to have multiple protective effects on the intestine and suppress inflammation, and can produce SCFAs (mainly propionic acid) [52, 54, 55], which precisely aligns with our findings, especially the strong positive correlation between *s_A. muciniphila* and propionic acid. Reduced levels of *A. muciniphila* are associated with immune diseases such as AA [56] and ulcerative colitis [57]. GPCRs play direct roles in protecting against colitis and controlling cytokine and chemokine production in intestinal epithelial cells, and among them, GPR41 is particularly activated by propionic acid [58–60]. propionic acid has been shown to inhibit TSLP expression and eosinophil proliferation [61] and promote tight junction protein expression [52]. It can also mitigate lung inflammation caused by asthma and enhance bronchial epithelial cell barrier function by regulating immune cells [62–64]. Therefore, the protective effect of BAI on the gut in AA mice is partly due to the increase in *A. muciniphila* and its metabolite propionic acid. Through FMT experiments, this study further demonstrated the successful colonization of the beneficial bacterium *A. muciniphila* from BAI donor faecal microbiota and the upregulation of GPR41 expression.

## Conclusion

**Fig. 8:**
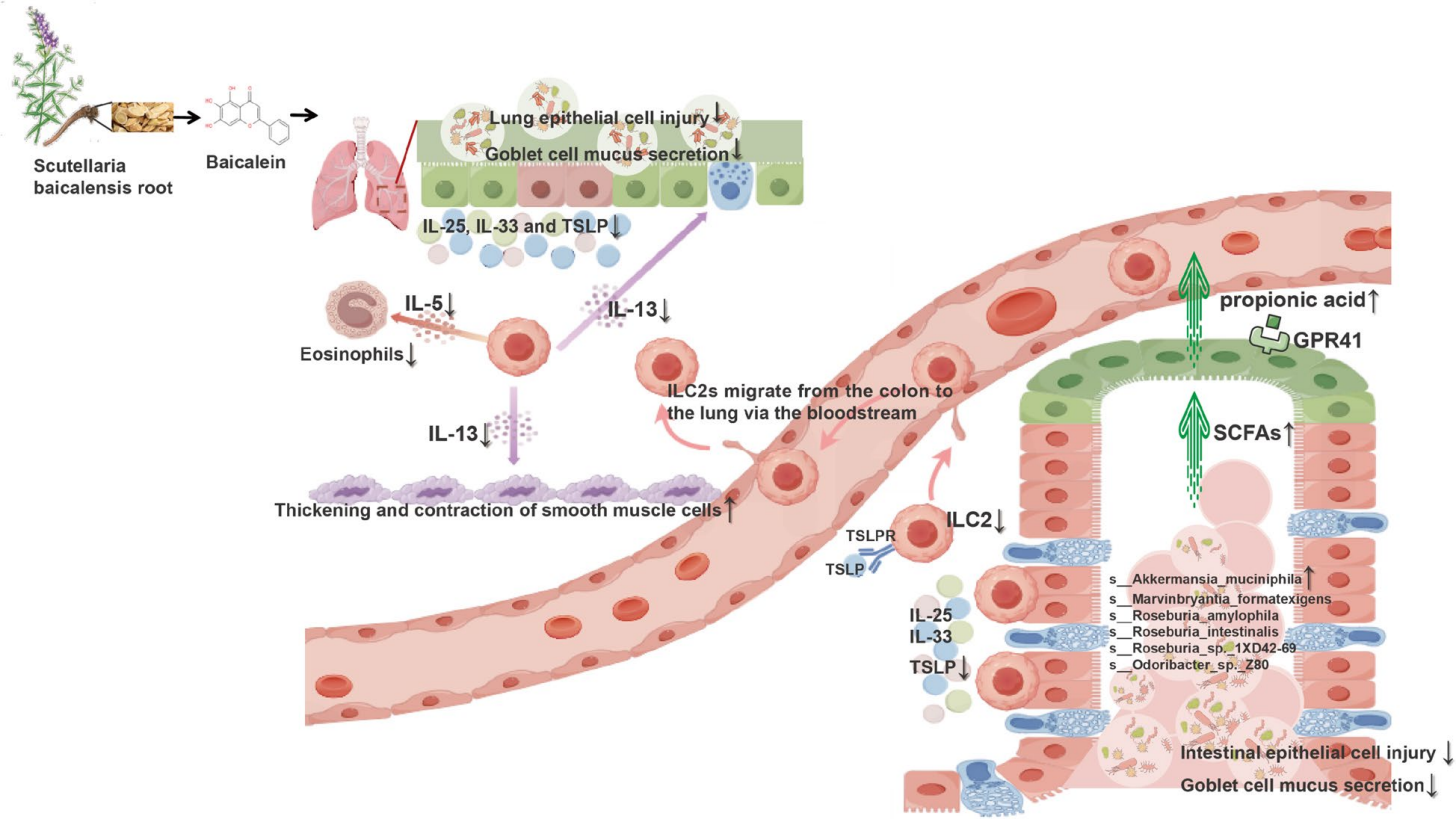
Proposed mechanism: BAI alleviates AA through microbiota dependent indirect pharmacological mechanism: enriching propionic acid producing bacterium *A. muciniphila* and activating GPCRs (especially GPR41), suppressing colonic epithelial barrier damage and ILC2-mediated immune activation, thereby further inhibiting lung epithelial barrier damage and type 2 immune responses. SCFAs, short-chain fatty acids; TSLP, thymic stromal lymphopoietin; TSLPR, TSLP receptor; IL-25, interleukin-25; IL-33, interleukin-33; IL-13, interleukin-13; IL-5, interleukin-5; ILC2s, type 2 innate lymphoid cells; GPR41, G-protein-coupled receptor 41

We propose an integrated mechanistic model for treating AA with BAI via the “gut–lung axis” (Fig. 8). On the one hand, BAI may act on the lungs through blood circulation, inhibiting its binding to receptors by binding to three alarmins, thereby enhancing the airway epithelial barrier, exerting local anti-inflammatory and microbial homeostasis maintenance effects. On the other hand, alleviating AA through microbiota dependent indirect pharmacological mechanisms enriches beneficial bacteria such as *A. muciniphila* and its representative metabolite propionic acid. This, in turn, may repair and maintain gut barrier integrity, prevent systemic low-grade inflammation triggered by “leaky gut,” and reduce the abnormal migration and production of gut-derived inflammatory cells (e.g., ILC2s) and cytokines. Future research could focus on whether specific bacterial strains (e.g., *A. muciniphila*) or their metabolites (e.g., propionic acid) alone can mimic part of the efficacy of BAI and how BAI precisely affects the migration and function of gut immune cells (e.g., ILC2s), thereby opening new avenues for developing precise intervention strategies targeting the “gut–lung axis”.

## Supplementary Information

Additional file1

## Data Availability

The data that support the findings of this study are available from the corresponding author upon reasonable request. The results of 16S rRNA sequencing have been uploaded http://www.ncbi.nlm.nih.gov/bioproject/1414244, BioProject ID: PRJNA1414244.

## Abbreviations

BAI: Baicalein
AA: Allergic asthma
SCFAs: Short-chain fatty acids
FMT: Faecal microbiota transplantation
*A. muciniphila*: *Akkermansia muciniphila*;
TSLP: Thymic stromal lymphopoietin
TSLPR: TSLP receptor
IL-25: Interleukin-25
IL-33: Interleukin-33
ILC2s: Type 2 innate lymphoid cells
WBP: Whole-body plethysmography system
TJ: Tight junction
ZO-1: Zonula occluden-1
