# Supplementary material for: Baicalein mitigates epithelial barrier impairment and microbiota dysbiosis in allergic asthmatic mice via the gut‑lung axis
Source: Chin Med. 2026 May 21;21:138. doi: 10.1186/s13020-026-01427-8 (PMC13191896; doi:10.1186/s13020-026-01427-8)
Supplement: Supplementary file 1 — Additional file1 [file 13020_2026_1427_MOESM1_ESM.docx]

Figure S1 The lung index. The data are expressed as the means ± SDs and were analysed by one-way ANOVA. *P < 0.05, **P < 0.01, ***P < 0.001 vs. the control group; #P < 0.05, ##P < 0.01, ###P < 0.001 vs. the model group.

Figure S2 The heatmap shows the changes in the pulmonary microbiota of mice in the control group, model group, and high-dose baicalein group at the genus level (n=6).

Figure S3 Molecular docking of BAI with 3 alarmins.


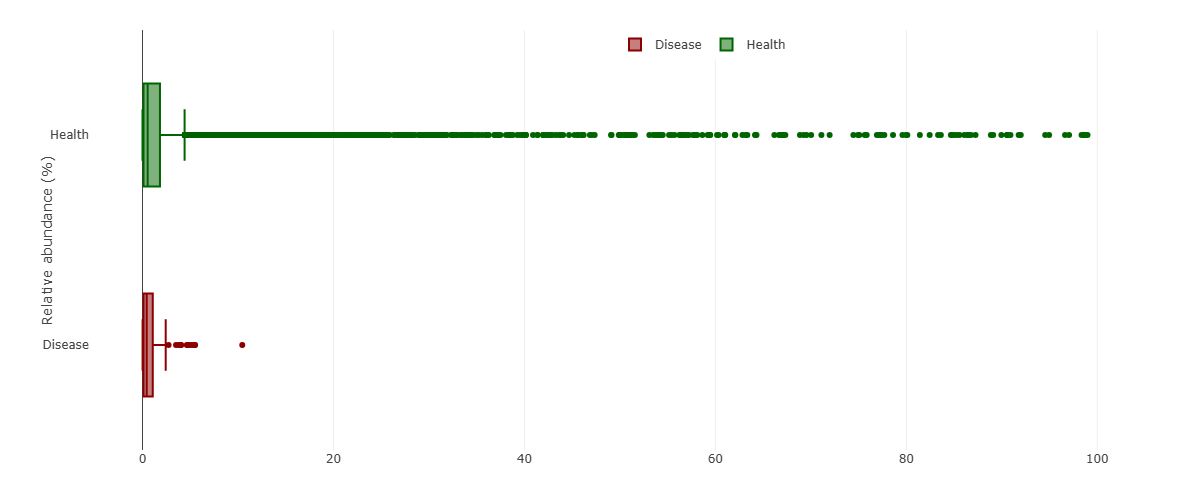


Figure S4 The abundance of A. muciniphila in feces of normal individuals and asthma patients in the GMrepo database.

Figure S5 Proportion of TSLPR in mouse colon tissue detected by flow cytometry. The data are expressed as the means ± SDs and were analysed by one-way ANOVA. *P < 0.05.

Figure S6 Proportion of TSLPR in FMT mouse colon tissue detected by flow cytometry. The data are expressed as the means ± SDs and were analysed by one-way ANOVA. *P < 0.05.
